# Holothurian Fucosylated Chondroitin Sulfate

**DOI:** 10.3390/md12010232

**Published:** 2014-01-09

**Authors:** Vitor H. Pomin

**Affiliations:** Program of Glycobiology, Institute of Medical Biochemistry Leopoldo de Meis, and University Hospital Clementino Fraga Filho, Federal University of Rio de Janeiro, Rio de Janeiro, RJ 21941-913, Brazil; E-Mail: pominvh@bioqmed.ufrj.br; Tel.: +55-21-2562-2939; Fax: +55-21-2562-2010

**Keywords:** carbohydrate-based drug development, chondroitin sulfate, fucose, sulfated glycosaminoglycan, echinoderm, therapeutic actions

## Abstract

Fucosylated chondroitin sulfate (FucCS) is a structurally distinct glycosaminoglycan found in sea cucumber species. It has the same backbone composition of alternating 4-linked glucuronic acid and 3-linked *N*-acetyl galactosamine residues within disaccharide repeating units as regularly found in mammalian chondroitin sulfates. However, FucCS has also sulfated fucosyl branching units 3-*O*-linked to the acid residues. The sulfation patterns of these branches vary accordingly with holothurian species and account for different biological actions and responses. FucCSs may exhibit anticoagulant, antithrombotic, anti-inflammatory, anticancer, antiviral, and pro-angiogenic activities, besides its beneficial effects in hemodialysis, cellular growth modulation, fibrosis and hyperglycemia. Through an historical overview, this document covers most of the science regarding the holothurian FucCS. Both structural and medical properties of this unique GAG, investigated during the last 25 years, are systematically discussed herein.

## 1. The First Reports Were Mostly Concerned with the Structure and Physicochemical Properties

The first description about the existence of the unique marine glycosaminoglycan (GAG) named fucosylated chondroitin sulfate (FucCS) is the work of Vieira and Mourão (1988) [1]. In this work, the authors have investigated the physical aspects and chemical compositions of three major fractions of sulfated polysaccharides isolated from the body wall of the sea cucumber *Ludwigothurea grisea* (Echinodermata, Holothuroidea). These fractions were obtained by separation based on net charge on a DEAE-column (an ion-exchange resin) [1]. While two of these fractions proved to predominantly contain sulfate groups and fucopyranosyl (Fuc*p*) units, the fraction of the highest yield was composed of approximately equimolar quantities of α-l-Fuc*p*, β-d-glucuronic acid (GlcA), and *N*-acetyl β-d-galactosamine (GalNAc) units. The structure of this fraction was further examined beyond the monosaccharide composition. This glycan proved to have a very unusual structure composed of a chondroitin sulfate (CS)-like backbone together with side-chain sulfated fucopyranosyl units linked to approximately half of the GlcA moieties through their *O*-3 position. The presence of these unusual fucosyl branches obstructed the accessibility of chondroitin sulfate-related digestive enzymes, named chondroitinases, which normally act on the regular CS cores. After partial acid hydrolysis, this core becomes susceptible to chondroitinase degradation because the sulfated fucose residues were not attached to the polymer. The digestion released both 6-sulfated and non-sulfated CS disaccharides [1]. This composition was later corrected as described below.

**Figure 1:**
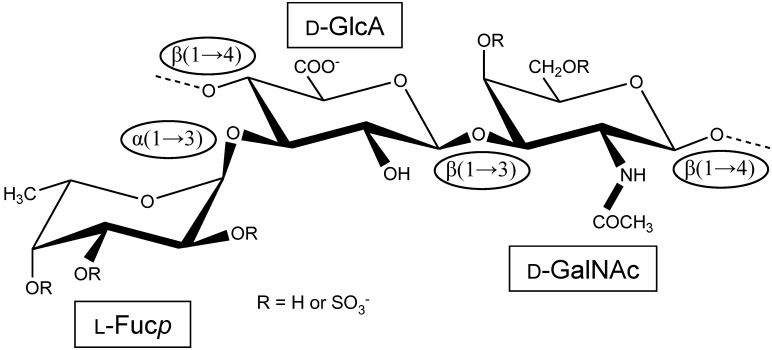
Structural representation of the repeating unit of the holothurian fucosylated chondroitin sulfate (FucCS). The monosaccharides are indicated by rectangles. They are α-l-fucose (l-Fuc*p*), β-d-glucuronic acid (d-GlcA), and *N*-acetylβ-d-galactosamine (d-GalNAc). The glycosidic linkage types are indicated in ellipses.

The structure of this same *L. grisea* FucCS molecule was deeper investigated in further work by the same group using a combination of chemical reactions like desulfation, defucosylation, and methylation together with nuclear magnetic resonance (NMR) spectroscopy analyses [2]. In this work, the authors unequivocally confirmed that this unique marine GAG contains side-chain sulfated Fuc*p* units linked to approximately one-half of the GlcA moieties through their *O*-3 position (Figure 1). Although in the previous work the authors observed that FucCS becomes more susceptible to chondroitinase degradations after defucosylation [1], it is only after an additional desulfation reaction that the FucCS becomes more easily degradable by the chondroitinases AC or ABC [2]. This result, together with methylation and NMR analyses, suggested that besides the fucosyl branches, the sea cucumber FucCS may also contain sulfate esters, which were determined to be at position *O*-3 of the beta-d-GlcA units. Although being an unusual type of sulfation, this feature was confirmed by the use of a specific monoclonal antibody that specifically recognizes the 3-*O*-sulfo-β-d-GlcA units. In analogy with the fucose branch units, this 3-*O*-sulfonation imposes some resistance to the digestive enzyme activities. Although a highly heterogeneous structure has been indicated in this work, the authors raised some suggestions about the major structural components of this marine GAG [2]. They concluded that the branches of *L. grisea* FucCS could be composed of disaccharide units of 3,4-di-*O*-sulfo-α-l-Fuc*p* units 2-linked to 4-*O*-sulfo-α-l-Fuc*p* units. This disaccharide conception was later corrected. The branches are currently accepted to contain just a single fucose residue (Figure 1). Finally, from this work [2], the CS-composing units were also corrected. Besides the commoner 6-sulfated and non-sulfated units, 4,6-disulfated GalNAc units were also detected from the CS core analyses after near-complete digestions [2]. These amounts of substituted GalNAc units were again later corrected as detailed below.

In reference [3], Vieira and coauthors changed their perspective of study, from the isolated FucCS molecule itself towards the macromolecular ensemble, which would be the proteoglycan form containing the FucCS chains attached. The authors observed that FucCS-bearing proteoglycans from the body wall of the sea cucumber *L. grisea* may be attached to protein cores of a multitude of molecular sizes, as opposed to mammal proteoglycan which are attached to a single type of protein core. Although varying in protein chain sizes, the FucCS-containing proteoglycans of *L. grisea* were shown to have a similar hexuronic acid/protein ratio and similar type of glycan chain structures [3]. Nevertheless, this reference was unequivocally the first report describing a structurally heterogeneous proteoglycans of the same kind, and from the same organism, in terms of protein composition.

Still concerning the physicochemical properties of FucCS, the following study from the same group of researchers, lead by Prof. Mourão, investigated the calcium binding properties and charge distribution of the *L. grisea* FucCS compared to other sulfated polysaccharides, such as chondroitin 6-sulfate (CS-C), and the sulfated fucan with the following structure [→3)-α-l-Fucp-2,4di(OSO_3_^−^)-(1→3)-α-l-Fuc*p*-(1→3)-α-l-Fuc*p*-2(OSO_3_^−^)-(1→3)-α-l-Fuc*p-*2(OSO_3_^−^)-(1→]*_n_*, also isolated from the sea cucumber *L. grisea* [4]. The FucCS was shown to have an approximately fivefold higher affinity for calcium ions than the standard CS-C. The authors have hypothesized that this increased affinity for calcium ions is likely due to the presence of the fucosyl branches, since the calcium affinity of the linear sulfated fucan was similar to that observed for the standard CS-C. More charged groups per disaccharide unit, and likely a shorter distance between these groups, make FucCS, sulfated fucan and CS-C very distinct from each other. Comparison between native and chemically modified (desulfated and carboxyl-reduced) derivatives have suggested that sulfate esters are responsible for the increased charge density of the FucCS, and that the presence of the fucose branches does not alter the length of the repetitive units in the CS core of the sea cucumber molecule [4].

In a paper primarily investigating the anticoagulant properties of FucCS, further insights into the structural aspects of this molecule were also provided [5]. After employing mild acid hydrolysis to remove the sulfated fucosyl branches, the authors characterized the released units by ^1^H NMR spectroscopy. The most abundant unit was 4-*O*-sulfo-α-l-Fuc*p*, although 2,4-di-*O*- and 3,4-di-*O*-sulfated units were detected as well. However, this data was again later corrected. Degradation of the remaining polysaccharide (the CS core) with chondroitin ABC lyase showed that the sulfated α-l-Fuc*p* units, previously released by mild acid hydrolysis, were significantly concentrated at the non-reducing end terminal of the backbone. This was concluded because the enzyme-resistant CS sequence includes the reducing terminal and carries acid-resistant-Fuc*p* substituted moieties [5]. The structural findings from this reference complemented the previous works [1,2,3,4], and showed that in structural characterization, a single work cannot be taken as definitive.

## 2. Medical Effects

### 2.1. Anticoagulation and Antithrombosis: The First and Predominantly Studied Clinical Actions

The concerns about the physicochemical aspects of FucCS make sense for the initial period of the FucCS research history. Besides leading the first achievements in this regard, the group of Prof. Mourão also pioneered studies on the possible therapeutic properties of the holothurian FucCS [5]. In the initial stages, they discovered the beneficial capacity of FucCS in blocking, depending on doses, the blood coagulation proteases [5]. Mourão *et al.* [5] demonstrated that the anticoagulant activity of the *L. grisea* FucCS is conferred by the presence of its sulfated α-l-Fuc*p*-made branch units. This conclusion comes from the fact that the anticoagulant activity of the *L. grisea* FucCS is lost upon defucosylation or desulfation reactions. The lack of anticoagulant activity in the mammalian CS, which is naturally unfucosylated, supports the importance of such fucosyl branches to the anticoagulation. The anticoagulant activity of FucCS was observed by its capacity for prolonging the clotting time, measured by the activated partial thromboplastin time (aPTT) assay. The anticoagulant activity of the FucCS from *L. grisea* was shown to be much greater than that of the sulfated fucan isolated from the same organism (structure described above), even though both structures show similar sulfation levels [5]. In addition, assays using purified coagulation cofactors have shown that FucCS can potentiate thrombin (IIa) inhibition via both antithrombin (AT) and heparin cofactor II (HCII) activities [5].

Besides the anticoagulant effects [5], FucCS has also been demonstrated to exhibit properties against thrombosis [6,7,8,9,10,11,12,13,14,15,16,17]. Since tromboembolic diseases are a major cause of deaths in the world, studies about the antithrombotic potentials of FucCS have been the leading research program so far. The first description about the possible antithrombotic activity of FucCS was obtained again from studies using the molecule extracted from *L. grisea* [6]. In reference [6], the FucCS, and its chemically modified derivatives were assayed using a stasis thrombosis model in rabbits. Intravenous administration of the native FucCS has been shown to reduce thrombosis in a dose-dependent manner, and specifically at a dose of 1.5 mg/kg (60 IU/kg) body weight, it completely prevented thrombosis after 10 min stasis. After intravenous injection of the antithrombotic dose of radioactively labeled FucCS, an inverse correlation was observed between removal of radioactivity from the plasma and decrease in *ex vivo* aPTT values, demonstrating that antithrombotic effectiveness depends on the level of circulating polysaccharide rather than on an indirect effect of the marine glycan on the vascular endothelium. Reduction of the GlcA carboxyl groups in FucCS by carboxyl-reduction reaction was shown to not affect the *in vitro* and *in vivo* FucCS activities. Partially defucosylated and desulfated derivatives have no anticoagulant or antithrombotic activities. Similar to what has been shown in reference [5], the removal of the sulfated fucosyl branches of the holothurian polysaccharide greatly abolished the antithrombotic effect [6]. These results support again the importance of the fucosylation on the antithrombotic activity of the marine GAG [6].

In the work of Pacheco *et al.* [7], the authors compared the antithrombotic activity of *L. grisea* FucCS with those of standard mammalian GAGs like unfractionated heparin (UFH), low molecular weight heparin (LMWH) and dermatan sulfate (DS). Intravascular injection of FucCS in a dose that totally prevents the thrombus formation has shown the ability to produce a much more intense modification of the plasma anticoagulant activity than antithrombotic doses of UFH, LMWH and mammalian DS. From this observation, the authors postulated that the antithrombotic mechanisms of action of these sulfated GAGs must be different. For the holothurian FucCS, it depends mostly on modifications of the plasma anticoagulant activity. The anticoagulant and possibly the antithrombotic actions of FucCS were described to be mostly dependent on HCII activity. Both actions were observed to markedly reduce with the decreasing of the chain size of the polymers. The holothurian FucCS revealed also an evident effect in preventing experimental venous thrombosis [7].

In the work of Zancan *et al.* [11], the authors have compared the anticoagulant, bleeding and antithrombotic effects of the *L. grisea* FucCS with its carboxyl-reduced derivative by *in vivo* experiments in rats. Both compounds have shown similar anticoagulant activity, mostly due to acceleration of thrombin inhibition in the presence of HCII. This data has pointed again to the minor effect of the carboxyl groups on the FucCS biological action. The native FucCS has shown a good correlation between anticoagulant, bleeding and antithrombotic effects. Inhibition of thrombosis in an arterial-venous shunt model occurs solely at low doses, and these doses were observed almost ineffective in modifying the anticoagulant activity of the plasma. In a venous experimental model, on the contrary, antithrombotic activity requires high doses and occurs concomitantly with an increased anticoagulant activity of the plasma [11]. The action of *L. grisea* FucCS on thrombosis is apparently unrelated to its effect on platelet aggregation [11]. The carboxyl-reduced derivative of FucCS prevented thrombosis in the arterial-venous shunt model, but not in the venous experimental model. This derivative did not increase bleeding, despite its potent anticoagulant effect. From these data, besides dissociating anticoagulant, bleeding and antithrombotic effects, the authors have also demonstrated that there is great possibility for generating a polysaccharide that is a potent inhibitor in arterial thrombosis, but inactive in venous thrombosis [11]. This follows the rationale in design for antithrombotic agents with specific mechanisms of action.

A great achievement on the pharmaceutical properties of FucCS against thrombosis was the discovery that the antithrombotic action of FucCS can be retained when this marine compound is orally up-taken [12]. The fact that intravascular injections of FucCS have the capacity to inhibit thrombus formation in the arterial shunt model in rats [11] has raised the hypothesis that this compound might be resistant to digestion by certain enzymes in low pH in the stomach. To test this hypothesis, the possible antithrombotic therapeutic action of *L. grisea* FucCS after oral administration was investigated. Indeed, when orally administrated into rats, a dose-dependent increase in the plasma anticoagulant activity of FucCS was observed. This was verified by assays of aPTT, thrombin time (TT) (about three and fivefold, respectively), and by anti-IIa activity in animals that received daily oral doses of the holothurian FucCS. These FucCS-administrated animals had a decrease in thrombus weight based on both experimental models of venous and arterial shunt thrombosis. This antithrombotic action was shown to have a strong positive relationship with anticoagulant activity. Similar doses of UFH administered orally had no effect on the plasma anticoagulant activity, neither on the thrombus weight. The authors also observed that when the holothurian FucCS is given orally to rats, it shows no capacity to modify the bleeding time. This reference has demonstrated that FucCS can be active and absorbed after oral administration, and these achievements greatly support the development of a new carbohydrate-based anticoagulant and antithrombotic agent that can be orally up-taken [12].

This orally absorption and clearance of FucCS was previously pointed out by the work of Imanari *et al.* in 1999 [18]. Partially depolymerized holothurian glycosaminoglycan (DHG) was administered by both intravenous and oral routes in experimental animals. After intravenous injection, clearance of DHG, as measured by postcolumn high pressure liquid chromatography (HPLC), displayed complex kinetics that were not dose dependent. DHG was excreted unchanged in the urine of the animals, thus revealing a certain type of metabolic resistance. No degradation products of DHG were detected by either gel filtration or anion exchange HPLC at any time in plasma, indicating that administered DHG is not catabolyzed by mammal metabolism. Anion exchange chromatographic behavior of DHG excreted into urine after oral administration has shown that partial desulfation might occur through intestinal absorption. After oral administration of DHG (50 mg/kg), 0.1% of the dose was found in urine collected for 24 h. More than 5% of intravenously administered DHG (1 mg/kg) was excreted into urine in 24 h. These results have suggested that orally administered macromolecules of high molecular weights, such as DHG, can be absorbed in the gastrointestinal tract [18]. The molecular weight of DHG was determined by another work, and proved to be 14 kDa [19]. This lifetime of circulating DHG in plasma after oral administration is controlled mainly by its renal clearance rather than catalytic metabolism [18].

The relevance of the fucosyl branched units to the anticoagulant and antithrombotic actions of FucCS has been clearly pointed out previously [5,6]. For the best response of these activities, however, not only the presence of these branches is important, but actually the presence and percentage of the 2,4-disulfated fucosyl units [14]. This conclusion comes from a comparative study of the effects of two 2,4-disulfation-rich sulfated polysaccharides on coagulation, thrombosis and bleeding. They were the FucCS and the sulfated fucan, both extracted from the sea cucumber *L. grisea*. While the 2,4-disulfated Fuc*p* unit is just a composing constituent of the backbone in the sulfated fucan, in holothurian FucCS, it is a branching unit [5]. The anticoagulant activities of the sulfated fucan and the FucCS were measured to be 10 and 55 IU/mg, respectively, by the aPTT method using a 229 IU/mg international UFH standard (Table 1). These anticoagulant potentials seem to be proportionally related to the amounts of the 2,4-*O*-di-sufated Fuc*p* units in these polymers [14].

**Table 1:** Sulfation patterns (proportions of the branching sulfated fucose units) and the anticoagulant potential (measured by aPTT) of FucCS from 12 sea cucumber species analyzed so far.

Species	Fuc0S	Fuc3S	Fuc4S	Fuc2S4S	Fuc3S4S	aPTT	References
*Ludwigothurea grisea* ^a^	0	−	~49	~20	~17	55^ b^	[5,14]
*Pearsonothuria graeffei*	−	−	81.6	18.4	−	35 ^c^	[20]
*Holothuria vagabunda*	25.6	−	50.2	15.8	8.4	42^ c^	[20]
*Stichopus tremulus*	−	−	24.8	22.4	52.8	135 ^c^	[20]
*Isostichopus badionotus*	−	−	4.1	95.9	−	183 ^c^	[20]
*Thelenata ananas*	0	~25	~22	~53	0	348 ^d^	[21,22]
*Stichopus japonicus* ^e^	0	Nd ^f^	11.1	55.6	33.3	Ns ^g^	[23]
*Holothuria edulis* ^h^	−	−	Nd	18	Nd	89 ^i^	[24]
*Apostichopus japonicas* ^h^	−	−	Nd	45	Nd	116 ^i^	[24]
*Holothuria nobilis* ^j^	−	Nd	Nd	−	Nd	59 ^i^	[24]
*Acaudina molpadioidea* ^k^	−	−	−	−	−	Nc ^l^	[25]
*Athyonidium chilensis* ^k^	−	−	−	−	−	Nc ^l^	[26]

^a^ The CS backbone of FucCS from *L. grisea* has been extensively characterized. It is composed of GalNAc units with the following substitution percentages: 12% 4,6-di-sulfated, 53% 6-mono-sulfated, 4% 4-mono-sulfated, and 31% non-sulfated [14]. ^b^ aPTT values expressed as international units/mg (IU/mg) using a parallel standard curve based on the International Heparin Standard (UFH) whose activity is 229 units/mg [14]. ^c^ aPTT values expressed as international units/mg (IU/mg) using a parallel standard curve based on the International Heparin Standard (UFH) whose activity is 150 units/mg [20]. ^d^ aPTT values expressed as international units/mg (IU/mg) using a parallel standard curve based on the International Heparin Standard (UFH) whose activity is 204 units/mg [21]. ^e^ The CS backbone of this FucCS was mostly characterized as CS-E [27], which is predominantly composed of 4,6-*O*-di-sulfated GalNAc units. ^f^ Not determined. ^g^ Not studied. ^h^ Although the mono-4*S* and di-3*S*4*S* fucosyl units have been assigned in the FucCS of *H. edulis* and *A. japonicas* in [24], the amounts of these units were not provided therein. ^i^ aPTT values expressed as international units/mg (IU/mg) using a parallel standard curve based on the International Heparin Standard (UFH) whose activity is 212 units/mg [24]. ^j^ The FucCS from *H. nobilis* was studied by NMR but the anomeric signals belonging to the fucose residues were rather to weak and broad to allow integration and further quantitation of their proportions. However, mono-3*S*, mono-4*S*, and di-3*S*4*S* fucosyl units were clearly observed [24]. ^k^ Structures studied by Fourier transformed-infrared spectroscopy. Just a few structural features were raised. The sulfation patterns of FucCS from these two holothurian species are still an unknown. ^l^ Not clear. Although the aPTT assay was undertaken and values were measured for different FucCSs concentration, the final values in IU/mg in comparison with a standard UFH curve were not provided.

In the work of Chen *et al.* [20], the investigators have structurally characterized (Table 1) and measured the anticoagulant activity of FucCS molecules from four sea cucumber species. They were *Pearsonothuria graeffei* (from Indo-Pacific), *Stichopus tremulus* (from Western Indian Ocean), *Holothuria vagabunda* (from Norwegian coast), and *Isostichopus badionotus* (from Western Atlantic). NMR analyses have indicated three different sulfation patterns on the fucosyl branches (Table 1): 4-*O*-mono, 2,3-*O*-di, and 2,4-*O*-di-sulfation, all differently distributed in the four FucCS species [20]. The assessment and comparison of their anticoagulant activities have also shown that the species containing higher amounts of the 2,4-*O*-di-sulfated Fuc*p* unit, specially the FucCS from *I. badionothus* which is composed of 95.9% of this unit, are in fact more anticoagulant. The anticoagulant activity of this FucCS was measured to be 183 IU/mg by the aPTT method (Table 1) using a 150 IU/mg international UFH standard [20].

The 2,4-*O*-di-sulfation requirement for the anticoagulant activity was again raised in the work of Wu [21]. By NMR analyses, the structure of the FucCS (Table 1) from *Thelenata ananas* was fairly characterized. It showed a distribution of 3-*O*-mono, 4-*O*-mono, and 2,4-*O*-di-sulfation pattern in a proportion of 25%:22%:53%, respectively. Therefore, half of the fucosyl residues in this FucCS is 2,4-di-sulfated. The anticoagulant activity of this species was measured to be 348 IU/mg by the aPTT method using a 204 IU/mg international UFH standard [21]. These last three works [14,20,21] have demonstrated the crucial contribution of the 2,4-*O*-di-sulfated fucosyl branching units of FucCS to the success of its anticoagulant and antithrombotic activities.

The molecular size influence on the anticoagulation has also been pointed out [22]. Low molecular weight fractions were produced from FucCS of *T. ananas* by free-radical depolymerization [22]. The authors have concluded from this work that for prolonging the aPTT activity, an oligosaccharide chain of at least 6–8 units (6–7 kDa) is required for *T. ananas* FucCS.

A parallel between the anticoagulant and antithrombotic activities of the holothurian FucCS and the oversulfated chondroitin sulfated (OSCS), the contaminant of the UFH batches, have been established recently [15]. Both CSs have shown both serpin-independent and -dependent anticoagulant activities. However, those actions differ significantly considering the target coagulation protease and preferential serpins (AT or HCII). When these CSs were tested using a venous thrombosis experimental model, the holothurian FucCS is more potent as an antithrombotic agent than the OSCS. Both CSs activated factor XII in *in vitro* assays, based on kallikrein release [15].

FucCS is generally recognized for its high anticoagulant and antithrombotic activities [5,6,7,8,9,10,11,12,13,14,15,16,17]. It was mostly thought that these activities were essentially driven by a serpin-dependent mechanism in which mostly of the thrombin (or factor Xa) activities would be inhibited by HCII, or AT inhibitors. However, a new trend for explaining these therapeutic activities has been rising. This new trend is based on the serpin-independent mechanism data [27,28,29,30]. This mechanism has been raised from the fact that anticoagulant effects can be reached by FucCS in experiments using AT- and HCII-free plasmas. This unusual effect was obtained in assays using the *L. grisea* FucCS [28], as well as DHG obtained from *S. japonicus* [27,29,30]. In the work of Nagase *et al.* [27], DHG which had been partially characterized by Yoshida and Minami in 1992 [23], was obtained and tested by the aPTT method using normal, AT-depleted, HCII-depleted and doubly AT/HCII-depleted plasmas. Other tests like thrombin clotting time (TCT) and measurements of the activations of factor X by factor IXa, factor X by factor VIIa-tissue factor (TF), and AT or anti-factor Xa chromogenic assays were also undertaken [27]. The results indicated the DHG has two clear different mechanisms. One is the HCII-dependent thrombin inhibition, and the other is a serpin-independent inhibition [27]. After these findings, interference on the functions of factors VIII and V was also noted for DHG [31]. The serpin-independent anticoagulant action was shown to be mainly driven by the inhibition of the intrinsic tenase complex [30]. In addition to anticoagulation, the antithrombotic action of DHG was also demonstrated to be mainly driven by the serpin-independent mechanism [29], in which the inhibition of the intrinsic tenase complex formation predominates [30].

The therapeutic antithrombotic activity of DHG from *S. japonicus* has been proposed, with less bleeding effects than those observed for the gold standards UFH and LMWH [32]. This was also observed for the FucCS of *L. grisea* [11]. This phenomenon of lower bleeding effects associated with DHG than UFH has been further investigated by Minamiguchi and coauthors [33]. To characterize this difference, the authors examined the affinity of DHG for plasma proteins by means of a GAG-conjugated cellulofine column in comparison with that of UFH. The DHG column strongly bound factor V, factor IX, protein S, histidine-rich glycoprotein, platelet factor 4 (PF4), beta-thromboglobulin, von Willebrand factor, fibronectin, and HCII, but did not bind fibrinogen, prothrombin, factor VII, protein C, AT, plasminogen or alpha 2-plasmin inhibitor. The profile of protein binding to the UFH column was noted almost the same as that of the DHG column except that AT showed higher affinity for UFH. One of the reasons why DHG caused much less bleeding than UFH is thus suggested by the authors to be the differences in their affinity for AT in plasma [33]. The prolonged bleeding time caused by UFH and LMWH are also associated with the inhibition of thrombin-induced platelet aggregation, while DHG causes less bleeding than UFH and LMWH because of its different inhibiting activity on thrombin-induced platelet aggregation [34].

Similar to the clinical procedure of UFH neutralization routinely done in heparin-treated patients, the action for stopping the DHG anticoagulant activity was also studied using the proteins protamine sulfate and PF4 [35]. Given that the prolongation of bleeding time by DHG can be completely neutralized by protamine sulfate with concomitant normalization of TCT, and because thrombin inhibition activity of DHG can be neutralized by PF4, these two proteins can be safely used as antidotes for DHG to prevent the bleeding effect in case of an overdose [35].

Other studies using the DHG from *S. japonicus* have been focused on the measurement of its antithrombotic potential associated with variations of plasma concentrations after intravenous administration [36], as well as in measuring its anticoagulant effect via tissue factor pathway inhibitor (TFPI) by *in vivo* and *in vitro* experiments [37]. From this latter work, a new anticoagulant mechanism of action for DHG was raised. This mechanism comprises the inhibiting activity via the extrinsic pathway of the blood coagulation. The authors have concluded that the anticoagulant activity of DHG is based on various stages of the coagulation reactions. One would be inhibiting the initial steps of the extrinsic pathway. Another would be involving the intrinsic tenase complex, as also pointed out by the work of Sheehan and Walke [30]. The last one would be in the serpin-dependent thrombin inhibition mostly driven by the HCII activity [27].

Comparative structure-anticoagulant activity analysis of FucCS molecules from new holothurian species has recently emerged [24]. Three types of polysaccharides from the body wall of three sea cucumber species—*Holothuria edulis*, *Apostichopus japonicas* and *Holothuria nobilis*—were examined in regard to their physicochemical properties and anticoagulant activities (Table 1). Chemical composition and NMR analyses have indicated that two types of sulfated polysaccharides, sulfated fucan and FucCS, are found in all of the three examined species as previously described for *L. grisea* [1]. The FucCS molecules of these three species, although studied by NMR, were just partially characterized (Table 1). Besides FucCS and sulfated fucan, a neutral glycan was additionally observed in *H. edulis*. The FucCS molecules have shown stronger anticoagulant activities than the sulfated fucans, despite their molecular sizes being lower than those of the sulfated fucans. The neutral glycan has no activity as expected based on the absence of sulfation [24].

Other recent works have partially analyzed the structures and measured the anticoagulant activities of FucCS of two other holothurian species, *Acaudina molpadioidea* [25], and *Athyonidium chilensis* [26] (Table 1). The anticoagulant activities of the *N*-deacetylated, and deaminative depolymerized fragments of the FucCS from *T. ananas* were also recently studied [38]. Based on this latter work, the authors have indicated that although the anticoagulant activity of this FucCS species may be weaker when decreasing their molecular weights, no significant effects on intrinsic tenase-mediated factor X and HCII-mediated thrombin inhibitions can be noted [38]. This is a very relevant achievement if we consider the rational for designing novel anticoagulant agents, especially those which have been chemically modified to express unique and specific mechanisms of action.

### 2.2. Hemodialysis

Based on the knowledge that DHG from *S. japonicus* has anticoagulant properties, two works by Minamiguchi and coauthors have extended the therapeutic utility of this molecule for hemodialysis [39,40]. Through an anticoagulant experimental beagle-dog hemodialysis using a hollow-fiber dialyzer, the DHG activity was compared to that using UFH, LMWH, and nafamostat mesilate (FUT). Effectiveness was based on 5 h hemodialysis, and no marked clot deposition in the extracorporeal circuit was observed. At effective doses, UFH and LMWH significantly prolonged template bleeding time, in sharp contrast to FUT and DHG, which scarcely prolonged bleeding time during hemodialysis. DHG increased aPTT values about six times that of normal plasma and increased TCT markedly, while FUT resulted in marked aPTT increases but barely changed the TCT values in the hemodialysis circuit at its effective dose. The anticoagulant profile of DHG appears to be completely different from that of FUT. These results have suggested that DHG may be useful as anticoagulant for hemodialysis with low hemorrhagic risk [39]. The concerns about safety using the DHG in hemodialysis has been addressed in a later work using a dog model of renal failure in which all the above-mentioned compounds were again comparatively examined [40]. In this work, the authors have suggested the AT-independent anticoagulant activity of DHG to explain the low hemorrhagic risk of this FucCS compared to the standards heparins.

### 2.3. Atherosclerosis

The high-affinity of FucCS with lipoproteins has pointed towards a potential activity in atherosclerosis [41]. The authors of this work have seen a positive correlation between molecular weight of *L. grisea* FucCS and their binding affinity to LDL (low-density lipoprotein). This observation comes from a binding assay using fractionated FucCS chains of varying lengths. The binding affinity to LDL of these different fractions was observed to rise proportionally with the increase of the FucCS chain lengths. In comparison with other sulfated polysaccharides, FucCS has shown the greatest affinity to LDL. The following affinity increasing order was observed: vertebrate CS < *L. grisea* sulfated α-l-fucan < mammalian heparin < *L. grisea* FucCS [41]. Among many lipoproteins, FucCS has higher affinity for the apo B-containing lipoproteins like LDL and VLDL (very low-density lipoprotein). When sulfation or fucosylation are removed by desulfation and defucosylation reactions, the holothurian GAG loses its lipoprotein affinity. In this work, the authors have discussed the possible clinical contribution of FucCS regarding its high affinity for lipoproteins. They speculate that since sulfated polysaccharides are usually used in laboratories for determination and estimation of HDL (high-density lipoprotein)-cholesterol and LDL-cholesterol, because of their ability to form insoluble complexes with apo B100-containing lipoproteins in the presence of divalent cations, FucCS could be the best candidate among these sulfated polysaccharides, mostly due to its higher affinity property for lipoproteins. The amounts of these sulfated polysaccharide-lipoprotein-cholesterol complexes can be used as indicator of risks for coronary artery diseases in a prognostic perspective. As a compound that precipitates LDL and that can be obtained on a large scale from a single tissue and without significant structural variations like in mammalian heparins, the holothurian FucCS could be successfully used to generate a more accurate index in this diagnostic approach. The authors from this reference have even raised the possibility of FucCS usefulness in LDL-apheresis. This process concerns the plasma LDL clearance from hypercholesterolemic patients [41]. Curiously, although this reference has clearly shown the affinity of the holothurian FucCS with lipoproteins, no further investigations have been done in this field since its year of publication (1996).

Studies with the DHG from *S. japonicus* have also pointed to a possible beneficial function in atherosclerosis. Igarashi and coauthors have shown that DHG is clearly capable of preventing neointimal formation in a balloon-injured rat carotid artery experimental model [42]. The results from this work have indicated that DHG has an inhibitory effect on neointimal thickening induced by balloon catheterization, and this effect must be due to the inactivation of aberrant smooth muscle cells (SMC) by DHG.

### 2.4. Cellular Growth, Angiogenesis and Fibrosis

In order to further investigate the potential therapeutic use of FucCS from *L. grisea* as an antithrombotic agent, its effects on vascular SMC proliferation and endothelial cell proliferation, migration, and TFPI release was examined [43]. The experiments from this work were performed on SMC from rat thoracic aorta and on human umbilical vein endothelial cell (HUVEC) in culture with or without added fibroblast growth factors (FGF-1 and FGF-2). FucCS had a strong inhibitory effect on SMC proliferation, for which the IC_50_ was measured at 10 ± 5 μg/mL. FucCS showed no effect on HUVEC proliferation and migration assays in the absence of exogenous FGF, while heparin had clear inhibitory effects. FucCS at the dose of 10 μg/mL enhanced FGF-1- and FGF-2-induced HUVEC proliferation by 45% and 27%, respectively. In FGF-induced HUVEC migration assays, FucCS at a concentration of 10 μg/mL showed a strong enhancing effect with FGF-1 (+122%), which is around three times higher than that of heparin. FucCS also showed a lower enhancing effect with FGF-2 (+43%), whereas heparin had no effect. Finally, FucCS had the capacity for stimulating TFPI release, mainly in the free form (by 98%). Sulfated fucose branch groups were demonstrated to be essential to the effects on HUVEC proliferation and migration. Surprisingly, removal of the fucose branches did not abolish the TFPI release, as opposed to all other biological effects of FucCS analyzed so far. Partial reduction of the GlcA carboxyl groups limited the potentiating effect on HUVEC proliferation and migration, but did not affect TFPI release [43].

The knowledge about the effects of FucCS on cellular growth was extended by Tapon-Bretaudière *et al.* [44]. In this reference, they assayed *L. grisea* FucCS effects on endothelial cells through an *in vitro* angiogenesis model on Matrigel. FucCS, in the presence of FGF-2, showed a great effect for inducing the endothelial cells to form, in the Matrigel vascular tubes, with a well-organized capillary-like network and typical closed structures. Comparison between the activity of native and defucosylated and desulfated FucCS-derivatives has revealed that the sulfated fucose branches are the structural motif for the pro-angiogenic activity, like most of the biological actions tested for FucCS. As opposed to FucCS, heparin seemed to not induce angiogenesis in the experimental model [44].

Fibrosis is known to be an end point state for most renal diseases. Since GAGs have been shown to attenuate this process, in a similar fashion, the marine holothurian FucCS could be also active in the pathological states of renal fibrosis. Melo-Filho and coworkers (2010) have tested the *L. grisea* FucCS in renal fibrosis models of animals submitted to unilateral ureteral obstruction [45]. Animals were given 4 mg/kg body weight of FucCS intraperitoneally, once a day. After 14 days, their kidneys were examined by histological, immunohistochemical, and biochemical methods. Compared with control mice, collagen deposition decreased in the course of renal fibrosis in the animals receiving FucCS, as revealed by Sirius red staining and hydroxyproline content. The cellularity related to myofibroblasts and macrophages was also shown to be reduced, as was the production of transforming growth factor (TGF)-β. The total GAG content increased in the renal interstitium of animals submitted to unilateral ureteral obstruction compared with the control contralateral kidney, mostly due to an increased CS content. Interestingly, no change in the pattern of GAG deposition was observed after administration of the holothurian FucCS. Fibrosis induced by unilateral ureteral obstruction was attenuated in P-selectin-deficient mice, which did not respond to the invertebrate GAG as well. In conclusion, FucCS was fairly efficient in attenuating the renal fibrosis on a ureteral obstruction model in mice possibly through a P-selectin-mediated mechanism [45].

### 2.5. Tumor Metastasis and Inflammation

The most famous mammalian GAG, heparin, is an excellent inhibitor of P- and L-selectin binding to the carbohydrate determinant, sialyl Lewis(x) [46]. As a consequence of its anti-selectin activity, heparin attenuates metastasis and inflammation. The *L. grisea* FucCS has been tested for this property as well [46]. FucCS was demonstrated to be a potent inhibitor of P- and L-selectin binding to immobilized sialyl Lewis(x) and LS180 carcinoma cell attachment to immobilized P- and L-selectins. Inhibition occurred in a concentration-dependent manner. FucCS was four to eightfold more potent than heparin in the inhibition of the P- and L-selectin-sialyl Lewis(x) interactions. No inhibition of *E*-selectin was observed [46]. FucCS has shown the capacity to inhibit lung colonization by adenocarcinoma MC-38 cells in an experimental metastasis model in mice, as well as neutrophil recruitment in two models of inflammation (thioglycollate-induced peritonitis and lipopolysaccharide-induced lung inflammation). Inhibition was shown to occur at a dose different than that which produces change in plasma APTT. Removal of the sulfated fucosyl branches of the FucCS abolished the inhibitory effect *in vitro* and *in vivo*, in a similar way as described for the above-mentioned biological activities [5,6,7,41,44]. Besides the multitude of medicinal actions of FucCS, Borsig *et al.* have also suggested that this unique GAG from sea cucumber can be used as a potential alternative to heparin for blocking metastasis and inflammatory reactions without the undesirable side effects of anticoagulant heparin [46].

In addition to this last reference about the inhibiting activity of FucCS on selectin-mediated metastasis [46], recent studies have demonstrated another therapeutic mechanism of action for FucCS in cancer [17]. Since many cancers can occur concomitantly with thrombotic events, the dual beneficial effect of FucCS on thrombosis and cancer was evaluated [17]. The authors from this reference have demonstrated that the mouse melanoma B16F10 cells when treated with FucCS can display a significant reduction of metastasis and coagulation capacity, both *in vitro* and *in vivo*. Mechanistic studies revealed that treatment of B16F10 cells remarkably inhibited the formation of fibrin through attenuating the generation of activated factor Xa, without affecting the expression of both urokinase, and plasminogen activator inhibitor-1, which are the main two factors involved in fibrinolysis. Moreover, the invertebrate GAG treatment was shown to down-regulate the transcription and protein expression of TF. Promoter deletions, site mutations and functional studies have identified that the nuclear transcription factor NF-κB binding region is responsible for the FucCS-induced inhibition of TF expression. While the FucCS treatment of B16F10 cells was proved unable to inhibit NF-κB expression and phosphorylation, FucCS significantly prevented nuclear translocation of NF-κB from the cytosol, a potential mechanism underlying the transcriptional suppression of TF. Moreover, FucCS has markedly shown to also suppress the activation of p38MAPK and ERK1/2 signaling pathways, the central regulators for the expression of metastasis-related matrix metalloproteinases [17]. From this reference, FucCS was shown to exert a dual function in the inhibition of metastasis and coagulation activity in mouse melanoma B16F10 cells. Furthermore, this work has added significantly to the science of FucCS because it has supported the fact that the invertebrate fucosylated GAG may be indeed useful in therapies against cancer metastatic cases associated with blood disorders [17].

### 2.6. Viral Infection

Recently, it has been shown that the DHG may also express some antiviral activities, especially against HIV-strains [47,48]. In the work of Lian *et al.*, anti-HIV activities of *T. ananas* FucCS were assessed by a cytopathic effect assay and in a HIV-1 p24 detection assay [47]. The molecular interactions were explored via biolayer interferometry technology. The structure-function relationship was achieved by comparing its anti-HIV-1 activities, conserved CD4 induced (CD4i) epitope-dependent interactions, and anticoagulant activities. *T. ananas* FucCS was shown to efficiently and selectively inhibit the X4- and R5X4-tropic HIV-1 infections in C8166 cells with little cytotoxicity against C8166 cells and peripheral blood monocytes (PBMCs). The data from this reference has indicated that FucCS can bind to gp120 with nM affinity, and may interact with CD4i of gp120. Additionally, the CD4i binding affinity of FucCS was higher than that of dextran sulfate used as a positive control. Structure-function relationship studies have suggested that the unique sulfated fucose branches account for the anti-HIV-1 activity, like the other biological actions studied for FucCS. The molecular size and carboxyl groups of FucCS may also play important roles in various activities. FucCS-derivatives with modifications in these features have shown higher anti-HIV-1 activities and much lower anticoagulant responses than those of heparin and native FucCS [47]. This is promising for hemorrhagic HIV-infected cases.

In the work of Huang *et al.* [48], anti-HIV activities of the same *T. ananas* FucCS were evaluated for its capacity to block laboratory strain HIV-1_IIIB_ entry and replication (4.26 μg/mL and 0.73 μg/mL, respectively), in inhibiting infection by the clinic isolates HIV-1_KM018_ and HIV-1_TC-2_ (23.75 μg/mL and 31.86 μg/mL, respectively), and for suppressing HIV-1 drug-resistant virus [48]. From this reference, it has been shown that FucCS can also inhibit HIV-2_ROD_ and HIV-2_CBL-20_ replication (100 μg/mL). Notably, *T. ananas* FucCS has shown highly effective antiviral activity against T-20-resistant strains (EC_50_ values ranging from 0.76 μg/mL to 1.13 μg/mL). Further analyses have indicated that FucCS can potently bind to the recombinant HIV-1 gp120 protein, but no inhibition of recombinant HIV-1 reverse transcriptase was observed. From the work of Huang *et al.* [48], FucCS was shown to be capable of inhibiting several strains of HIV-1 replication with different levels of response. These results have suggested that FucCS may possess great potential to be further developed as novel HIV-1 entry inhibitor for treatment of HIV/AIDS patients, particularly for those infected by T-20-resistant variants. These two recent publications from Chinese scientists strongly support the development of new anti-HIV drugs from marine invertebrate sources [47,48].

### 2.7. Hyperglycemia

Recently, another study from Chinese researchers has demonstrated the beneficial effect of FucCS from the sea cucumber *A. molpadioides* to improve hyperglycemia of skeletal muscle from insulin-resistant mice [49]. In this study, FucCS, rosiglitazone (RSG), and their combinations were supplemented to a high-fat, high-sucrose diet (HFSD)-fed C57BL/6J mice for a period of 19 weeks. The results have shown that FucCS treatment has the capacity to decrease the blood glucose levels and insulin resistance. The glucose metabolism-related gene expression at the transcriptional level has also been measured, and apparently, they seemed to increase in skeletal muscle when FucCS is used [49]. Although the total protein expressions of IR-β, IRS-1, PI3K, PKB and GLUT4 in skeletal muscle were not affected, as measured by western blot assays, insulin-stimulated GLUT4 translocation and phosphorylation of Tyr-IR-β, Tyr612-IRS-1, p85-PI3K, Ser473-PKB, and Thr308-PKB were observed to significantly increase in cases in which FucCS was used as a supplement. A combination of FucCS and RSG was shown to produce synergistic effects on anti-hyperglycemia. The results from this study have clearly indicated that FucCS can alleviate hyperglycemic conditions via activation of the PKB/GLUT4 signaling pathway in skeletal muscle of insulin resistant mice [49]. This is a great reference for indicating the therapeutic activity of the holothurian FucCS in case of deficiencies related with glucose metabolism.

## 3. Chemical Modifications and Synthesis

Besides the already mentioned defucosylation, desulfation, and carboxyl-reduction reactions to produce FucCS-derivatives [5,6,7,41,43,44], *O*-acylation of FucCS fragments [50], and depolymerization procedures using ^60^Co irradiation [51] or free-radicals induced by hydrogen peroxide or copper ions [52] have also been employed. All these chemical reactions were undertaken to advance the structure-function relationship studies of FucCS. Since defucosylation, desulfation, and carboxyl-reduction reactions have been widely commented throughout this review, here we will be focusing solely on the latter three reactions: *O*-acylation of FucCS fragments [50], and depolymerization reactions using either ^60^Co irradiation [51] or free-radicals induced by hydrogen peroxide or copper ions [52]. These methods have not been discussed yet. A brief comment about chemical synthesis will also be added at this section.

In the work of Gao *et al.*, depolymerized FucCS samples were chosen for studies. They were submitted for selective substitution of their OH groups [50]. The *O*-acylation was carried out in a homogeneous way using carboxylic acid anhydrides. The structures of *O*-acylated FucCS fragments were extensively characterized by NMR. The results have indicated that the 4-*O*-sulfated fucose residues may be more easily acylated than the other sulfated fucosyl branching units. The *O*-acylation was always accompanied by β-elimination reaction, and the degree of elimination was observed to be higher when the acylation content was higher. The results of clotting assay have indicated that the effects of partial *O*-acylation on depolymerized FucCS samples and the *O*-acylation of 2-OH groups of 4-*O*-sulfated fucosyl branching units do not affect the anticoagulant activity of the derivatives [50].

The specific depolymerization method using ^60^Co irradiation in water solution was developed for the FucCS molecule isolated from the sea-cucumber species *P. graeffei* [51]. Fragments with varying molecular weights were obtained by this method employed at different dosages and sample concentrations. The chemical compositions and structures of these fragments were investigated based on HPLC, infrared, and NMR spectroscopy. The results have indicated that ^60^Co irradiation induced depolymerization via selective breakage of GlcA units in the *P. graeffei* FucCS backbone. No influence on the structural integrity of the sulfated fucose branches were seen under the mild conditions. The recommended conditions for the degradation of this FucCS were 2%–10% solution concentration and irradiation dosages of 10–50 kGy. The anticoagulant activities of the resultant low molecular weight fragments were additionally evaluated. The anticoagulant activities were significantly reduced with decreasing of the molecular weights, since the low molecular weight fragments have displayed significantly decreased anticoagulant activities when compared to the native *P. graeffei* FucCS [51].

In order to generate low molecular weight derivatives of FucCS from the sea cucumber species *T. ananas*, Wu *et al.* [52] have employed a process of depolymerization by free radicals. From this method, fractions with different and narrow molecular weight distributions were obtained. The parameters of the process were investigated in detail by HPLC size exclusion chromatography. The kinetics of the depolymerization process of *T. ananas* FucCS by H_2_O_2_ was established and the mechanisms of degradation have been proposed [52]. The results from this study have indicated that the levels of final products, fragmentation and reproducibility were different depending on the conditions of depolymerization used. The fragmentation of the main chain of *T. ananas* FucCS occurred randomly and obeyed pseudo-first-order kinetics. It has produced also fragments with rather narrow and unimodal distribution of molar masses. Chemical compositions of partially depolymerized samples were analyzed by ^1^H/^13^C-NMR spectroscopy and by elementary analysis. The results have suggested that there was no preferential cleavage of sulfated α-l-fucosyl branches, and chemical compositions of the products were kept almost unchanged when compared with the native *T. ananas* FucCS. This report has established a safe condition to depolymerize this specific FucCS without further changes in the structure [52]. Free-radical chemical depolymerization procedure of FucCS from *T. ananas* (incubated with copper (II) acetate monohydrate) was also successfully achieved by Wu and co-workers [22].

As the science of the holothurian FucCS has been evolving across the last 25 years, the backbone unit of FucCS containing the anticoagulant and antithrombotic active motif 2,4-*O*-di-sulfated fucosyl branch has recently been synthesized by chemical routes [53]. The structure of the FucCS trisaccharide obtained is the following (β-d-GalNAc-4,6di-(OSO_3_^−^)-(1→4)-β-d-GlcA-[(3→1)α-l-Fucp-2,4di-(OSO_3_^−^)]). The synthesis of this trisaccharide is itself a great accomplishment in the science of the holothurian GAG. Nonetheless, through the chemical polymerization of this unit, this science could go even further since this new achievement would allow the generation of a very relevant polysaccharide in terms of therapeutic properties.

## 4. Major Conclusions and Perspectives

FucCS is a unique GAG regarding its structure and medical properties. So far, it has been exclusively found in sea cucumber species (Echinoidea, Holothuroidea), in which it participates as a major extracellular matrix component of the body wall of this invertebrate. In terms of structure, regardless the holothurian species, FucCS is very similar to the mammalian CS, considering the backbone only. Both mammalian and invertebrate GAGs have the same composition of alternating 4-linked GlcA and 3-linked GalNAc residues in disaccharide repetitive units. However, the holothurian FucCS has also a branching Fuc*p* residue 3-linked to the GlcA unit (Figure 1). These Fuc*p* units have sulfation patterns that vary according to species (Table 1). These sulfation patterns were shown to account for different biological responses. For example, the sea cucumber species *L. grisea*, *T. ananas*, and *I. badionotus* which have FucCS-rich 2,4-di-sulfated Fuc*p* units, present high therapeutic effects on coagulation. The FucCS from *L. grisea*, *I. badionothus*, and *T. ananas* have 20%, 95.9%, and 53%, respectively, of 2,4-di-sulfated Fuc*p* units (Table 1). Their anticoagulant activities were measured to be 55, 279.4, and 389.3 IU/mg, respectively, by the aPTT method as compared, when normalized, to a 229 IU/mg international UFH standard [14,20,21]. Other FucCS species containing 2,4-*O*-di-sulfated fucose residues such as those from *H. vagabunda* and *P. graeffei* (15.8% and 18.4% of this unit, respectively, Table 1) have their anticoagulant activities as 64.1 and 53.4 IU/mg, respectively, as measured by the aPTT method as compared to a 229 IU/mg international UFH standard [20]. Hence, 2,4-disulfation in branching Fuc*p* units has been shown to be a crucial requirement for anticoagulation [14,20,21]. Although a fucosylated GAG, like FucCS, is itself a rare molecule in terms of structure, it is not unexpected to find it in holothurian species, since the marine invertebrates are equipped with the proper machinery to biosynthesize fucose-rich sulfated polysaccharides [54,55,56,57]. This conception can be supported by the fact that holothurian organisms are also able to express sulfated fucans as a component of their body walls, regardless of the species. The fucosyl branch in FucCS makes this molecule very distinct from the terrestrial GAG. Due to the presence of these sulfated fucosyl branching units, FucCS exhibits additional therapeutic actions, which the mammalian CS does not have. These potential therapeutic actions are anticoagulation, antithrombosis (including orally administrated), anti-HIV activity, improvement of hyperglycemic conditions, and beneficial effects in atherosclerosis, tumor metastasis, hemodialysis, cellular growth, angiogenesis, and inflammation. The branching Fuc*p* units play a key role in almost all these clinical actions, enabling the FucCS to be biologically active. Concerning the fields of carbohydrate-based drug discovery and development, the holothurian FucCS is certainly a special candidate. Table 2 summarizes in a straightforward way the major biological events in which the holothurian FucCS might be active, the mechanisms of action, structural requirements, and the assays used in the investigations.

**Table 2:** Biological events in which holothurian FucCS might be active, mechanisms of action, structural requirements, and assays used.

Biological Systems	Mechanisms of Action	Structural Requirement	Method	Reference
Coagulation/Thrombosis	Serpin-dependent action: FucCS potentiated the inhibition activity of blood cofactor antithrombin and heparin cofactor II over thrombin and factor Xa	Branch 2,4-*O*-di-sulfated Fuc*p* unit	*In vitro* TCT, aPTT, and tests using purified blood cofactors through chromogenic substrates.	[5,6,7,8,9,10,11,12,13,14,15,16,17,18,19,20,21,22,23,24,25,26,27,28,29,30,31,32,33,34,35,36,37,38]
			*In vivo* arterial and venous thrombotic models using mice and rats	
	Serpin-independent activity: FucCS inhibits formation of the intrinsic tenase complex besides interfering in the activity of factors VIII and V	Fucosyl branch units. The best sulfation pattern is still unknown	*In vitro* inhibitory assays using blood cofactors	
Hemodialysis	Anticoagulant activity	Fucosyl branch units since mammalian unfucosylated CS has no action in hemodialysis. The best sulfation pattern of FucCS branch units is still unknown	*In vivo* anticoagulant experimental beagle-dog method using a hollow-fiber dialyzer	[39]
			*In vivo* dog model of renal failure	[40]
Atherosclerosis	Interaction with lipoproteins	Fucosyl branch units. The best sulfation pattern is still unknown	Affinity liquid-chromatography	[41]
	Inhibitory activity over neointimal formation		*In vivo* balloon-injured rat carotid artery experimental model	[42]
Cellular growth	FucCS exhibits stimulating effects on vascular SMC proliferation and endothelial cell proliferation, migration	Fucosyl branch units. The best sulfation pattern is still unknown	*In vivo* assays using SMC from rat thoracic aorta and HUVEC in culture with or without added fibroblast growth factors (FGF-1 and FGF-2)	[43]
Angiogenesis	FucCS accelerates angiogenesis by interactions with FGF-2	Fucosyl branch units. The best sulfation pattern is still unknown	*In vitro* experiments for tubulogenesis using endothelial cells in Matrigel	[44]
Fibrosis	Inhibition of fibrosis via P-selectin-mediated mechanism	Fucosyl branch units. The best sulfation pattern is still unknown	*In vivo* renal fibrosis models of animals submitted to unilateral ureteral obstruction. Biochemical and histological analyses	[45]
Inflammation	Inhibitory activities over P- and L-selectins	Fucosyl branch units. The best sulfation pattern is still unknown	*In vitro* experiments using P- and L-selectin binding to immobilized sialyl Lewis(×)	[46]
Cancer metastasis	Inhibitory effects on selectin-mediated cancer metastasis	Fucosyl branch units. The best sulfation pattern is still unknown	LS180 carcinoma cell attachment to immobilized P- and L-selectins	[46]
Virus infection	FucCS binds to gp120 protein of HIV particles	Not assigned	*In vitro* cytopathic effect assay and a HIV-1 p24 detection assay (biolayer interferometry technology)	[47]
			*In vitro* inhibitory assays to verify blocking potential of FucCS on entry and replication of HIV strains	[48]
Hyperglycemia	FucCS enhances insulin-stimulated GLUT4 translocation and phosphorylation of Tyr-IR-β, Tyr612-IRS-1, p85-PI3K, Ser473-PKB, and Thr308-PKB	Fucosyl branch units. The best sulfation pattern is still unknown	*In vivo* experiments using skeletal muscle from insulin-resistant mice	[49]

Future tests utilizing FucCS oligosaccharides of well-defined molecular structures and weights in biological actions would allow prediction of the minimal length necessary for proper binding with the functional proteins involved in the therapeutic actions of FucCS. It is noteworthy also to investigate at the molecular and structural levels, the contribution of the fucose-composed branches on these therapeutic actions. These structural studies could be based on NMR, molecular modeling and molecular dynamics, using the polysaccharide free in solution or bound to proteins. It should be really fascinating not only for the science of FucCS, but also for glycosaminoglycanomics in general, to explain why a nearly inactive GAG, the CS, in many therapeutic actions like anticoagulation, antithrombosis, atherosclerosis, and viral infections, becomes active when bearing such sulfated fucose-containing branches. Maybe, the sulfate fucose might not only increase the electronegative charge density in FucCS polymers, but also lead to a drastic conformational change on FucCS so that a better interaction with the functional proteins can be reached. Another valuable perspective on this topic would be the polymerization of the (β-d-GalNAc-4,6di-(OSO_3_^−^)-(1→4)-β-d-GlcA-[(3→1)α-l-Fucp-2,4di-(OSO_3_^−^)]) trisaccharide recently synthesized by chemical routes [53]. This is the right anticoagulant and antithrombotic active motif of the holothurian FucCS. Although the chemical synthesis of this trisaccharide is itself a big achievement, the polymerization of this unit would comprise a real breakthrough. This polymerization procedure would allow the generation in laboratory and in large-scale of a potent anticoagulant and antithrombotic polysaccharide devoid of the major contaminant risks, such as those found in heparin, showing also higher activity, less bleeding effects and superior efficiency. This is perhaps the greatest perspective to be accomplished in the science of the holothurian GAG, and probably in glycomics if we consider the projects involved with carbohydrate-based drug development.
